# 2个罕见抗凝血酶和蛋白C联合缺陷家系的临床特征及基因分析

**DOI:** 10.3760/cma.j.cn121090-20250509-00219

**Published:** 2026-02

**Authors:** 跃丽 郭, 婷婷 单, 温洁莹 郑, 春 赵, 万仲 孔, 佩佩 金, 菁 戴

**Affiliations:** 1 上海交通大学医学院附属瑞金医院检验科，上海 200025 Department of Laboratory Medicine, Ruijin Hospital, Shanghai Jiao Tong University School of Medicine, Shanghai 200025, China; 2 浙江中医药大学附属温州市中医院检验科，温州 325000 Department of Laboratory Medicine, Wenzhou TCM Hospital of Zhejiang Chinese Medical University, Wenzhou 325000, China

**Keywords:** 静脉血栓栓塞, 联合缺陷, 抗凝血酶, 蛋白C, Venous thromboembolism, Combined defect, Antithrombin, Protein C

## Abstract

**目的:**

对2个抗凝血酶（AT）和蛋白C（PC）联合缺陷家系进行临床特征和基因突变分析，探讨SERPINC1基因和PROC基因联合缺陷与疾病发生的关系。

**方法:**

采用常规方法检测先证者及其家系成员AT活性（ＡT∶A）、AT抗原（AT∶Ag）、PC活性（PC∶A）、PC抗原（PC∶Ag）、蛋白S活性（PS∶A）指标。采用二代测序（NGS）和CNVplex技术对筛选基因的编码区和调控区进行点突变、小缺失或插入及基因拷贝数变异的检测，并对突变位点进行Sanger法测序验证。运用ClustalX-2.1-win软件分析突变位点的保守性；使用在线生物信息学软件预测突变位点的致病性；通过CAT法检测凝血酶生成情况。

**结果:**

先证者1的PROC基因第5外显子存在c.400+5G>A剪切位点突变，SERPINC1基因第5外显子存在c.883G>A（p.Val295Met）杂合错义突变；先证者2的PROC基因第9外显子存在c.811C>T（p.Arg271Trp）杂合错义突变，SERPINC1基因第5外显子存在c.880C>T（p.Arg294Cys）杂合错义突变，突变位点分别来源于患者的父亲和母亲。生物信息学分析发现以上突变位点大多数为保守位点，这些基因突变大多数被预测为“致病的、有害的”，可通过改变蛋白空间结构、破坏蛋白稳定性，引起AT和PC水平或功能异常。凝血酶生成试验结果显示，4例突变携带者均出现不同程度的内源性凝血酶生成增多呈高凝倾向，在血栓调节蛋白（sTM）的存在下，PC途径的抗凝功能明显减弱。

**结论:**

在2个家系中发现AT和PC两个突变位点，可能与2例患者的反复静脉血栓发生有关；SERPINC1和PROC基因联合突变携带者具有更高的静脉血栓发生风险。

## Abstract (en)

**Objective:**

To analyze the clinical characteristics and genetic mutations in two families with combined antithrombin（AT）and protein C（PC）deficiency, and to explore the relationship between combined SERPINC1 and PROC gene mutations and disease development.

**Methods:**

The AT activity（AT∶A）, AT antigen（AT∶Ag）, PC activity（PC∶A）, PC antigen（PC∶Ag）, and protein S activity（PS∶A）of the probands and their family members were measured. Next-generation sequencing（NGS）and CNVplex technology were used to detect point mutations, small deletions or insertions, and CNVs in the coding and regulatory regions of the selected genes, with mutation sites verified by Sanger sequencing. The conservation of mutation sites was analyzed using ClustalX-2.1-win software; the pathogenicity of these mutations was predicted using online bioinformatics tools; thrombin generation was assessed using the CAT method.

**Results:**

In proband 1, a c.400+5G>A splice site mutation was found in exon 5 of the PROC gene, along with a c.883G>A（p.Val295Met）heterozygous missense mutation in exon 5 of the SERPINC1 gene; in proband 2, a c.811C>T（p.Arg271Trp）heterozygous missense mutation in exon 9 of the PROC gene and a c.880C>T（p.Arg294Cys）heterozygous missense mutation in exon 5 of the SERPINC1 gene were identified. The mutation sites were inherited from the patient's father and mother, respectively. Bioinformatics analysis revealed that most of the mutation sites were conserved, and the majority of these gene mutations were predicted to be “disease-causing and harmful.” These mutations may lead to abnormal levels or functions of AT and PC by altering protein structure and destabilizing protein stability. Thrombin generation tests showed that all four mutation carriers exhibited varying degrees of increased endogenous thrombin potential, indicating a hypercoagulable state. In the presence of soluble thrombomodulin（sTM）, the anticoagulant function of the PC pathway was significantly impaired.

**Conclusion:**

Two families were found to harbor mutations in AT and PC genes, respectively, which may contribute to recurrent venous thromboembolism in the affected patients. Notably, individuals carrying combined mutations in the SERPINC1 and PROC genes have a significantly higher risk of venous thrombosis.

抗凝血酶（AT）是一种单链糖蛋白，主要由肝脏和血管内皮细胞合成，是人体内主要的生理性抗凝剂之一。AT主要灭活凝血酶和活化的凝血因子Ⅹ（FⅩa）从而发挥其强大的抗凝作用。在没有肝素的情况下，AT灭活凝血酶的效率处于生理水平，肝素的加入会诱导AT发生构象变化，从而使其抗凝活性显著提高[1]。蛋白C（PC）属于血浆中丝氨酸蛋白酶酶原家族，是一种在肝脏中合成的维生素K依赖性糖蛋白。经内皮细胞表面的凝血酶与血栓调节蛋白（TM）复合物激活后，成为活化蛋白C（APC），后者通过蛋白水解使活化的凝血因子Ⅴ（FⅤa）和活化的凝血因子Ⅷ（FⅧa）失活，从而抑制凝血级联反应中凝血酶的生成[2]。遗传性AT或PC缺陷症是由其编码基因SERPINC1或PROC突变引起的常染色体显性遗传疾病，患者的临床表现差异较大，与基因突变的数量、突变位点对蛋白功能的影响程度及基因多态性等密切相关[3]–[4]。本文对2个罕见的遗传性AT和PC联合缺陷家系进行了表型和临床分析，利用生物信息学软件初步探讨其分子发病机制及对凝血功能的影响。

## 对象与方法

一、研究对象

1. 家系1先证者：男性，外院诊断左下肢静脉、髂外静脉血栓形成（DVT），致肺栓塞（PE），为求进一步明确病因至上海交通大学医学院附属瑞金医院就诊。目前，予以口服抗凝药物利伐沙班15 mg每日两次及弹力袜治疗，病情稳定。家系其他成员否认血栓性疾病史。

2. 家系2先证者：男性，27岁时突发左下肢DVT，未予规范抗凝治疗；并在35岁时，出现左下肢DVT及PE，口服利伐沙班间断性抗凝治疗。本次突发胸闷，于上海交通大学医学院附属瑞金医院就诊，经CT确认PE，予以溶栓治疗，并置入滤网，低分子肝素（LMWH）常规剂量抗凝治疗2周，出院后服用利伐沙班20 mg每日1次维持治疗，目前病情稳定。家系其他成员否认血栓性疾病史。

3. 健康对照：为了建立实验室参考范围，从我院随机选取了100名健康受试者作为对照，以排除基因多态性，其中男性51名，女性49名，年龄38（22～46）岁。本研究已获得上海交通大学医学院附属瑞金医院伦理委员会批准（2019临论审54号），并且所有受试者均签署了知情同意书。

二、方法

1. 样本采集和凝血检测：采集研究对象外周静脉血2.7 ml，用0.109 mol/L枸橼酸钠溶液1∶9抗凝，1 500×*g*离心15 min后，取上层乏血小板血浆用于常规凝血指标的检测。在ACL-TOP全自动血凝仪（美国IL公司配套试剂盒）上采用发色底物法检测AT活性（AT∶A）和PC活性（PC∶A），采用凝固法检测蛋白S活性（PS∶A），采用酶联免疫吸附法（美国Enzyme Research Laboratories公司配套试剂盒）检测AT抗原（AT∶Ag）和PC抗原（PC∶Ag）。下层剩余的血细胞按照北京天根生化科技有限公司DNA提取试剂盒（离心柱型，批号Y2117）说明书提取基因组DNA。

2. 凝血酶生成试验（Thrombin Generation Test, TGT）：由于2例先证者均接受了抗凝治疗，不宜进行凝血酶生成试验，故我们采用2例先证者的父母（4个突变携带者）的乏血小板血浆进行凝血酶生成试验，用以评估突变对凝血酶生成的影响。使用经过校准的自动化凝血酶生成分析仪（法国Diagnostica Stago公司产品）按照说明书进行检测。试验通过添加含有5 pmol/L组织因子的商品化乏小板血浆试剂（法国Diagnostica Stago公司产品）启动反应，比较突变携带者血浆和健康对照血浆的凝血酶生成情况，并评价在可溶性重组人血栓调节蛋白（Soluble thrombomodulin，sTM，10 nmol/L）存在下PC途径的抗凝功能，所有样品均复孔检测。凝血酶生成曲线评估参数包括：凝血酶生成潜力（Endogenous thrombin potential，ETP），即凝血酶生成曲线下的积分面积，反映凝血酶生成总量；凝血酶生成峰值（Peak），为凝血酶生成的最大量；起始拖尾时间（STT），代表凝血酶生成的总时长。试剂盒及软件由Stago Thrombinoscope BV公司提供，所有操作步骤均严格按照试剂说明书进行操作[5]。

3. 基因检测：依据上海交通大学医学院附属瑞金医院检验科自主研发设计的易栓症基因检测Panel，共包含了35种与血栓发生相关的候选基因，采用二代测序和CNVplex技术对基因的编码区和调控区进行点突变、小缺失或插入及基因拷贝数变异（Copy number variation，CNV）的检测；从1 000个基因组数据库中删除dbSNP135中有记录的或中国人中等位基因频率≥1％的单核苷酸变异（Single nucleotide variant，SNV）；结合生物信息学手段对测序结果进行深度分析，进而确定相关基因的变异情况；最后根据二代测序结果，对筛查到的变异位点进行Sanger一代测序验证，同时对其家系成员的相同突变位点进行检测。

4. 生物信息学技术分析：应用多重序列比对软件ClustalX-2.1-win对人类和NCBI数据库中提供的其他同源性物种氨基酸突变位点的保守性水平进行研究（同源性物种的氨基酸序列来源网址：http://www.ncbi.nlm.nih.gov/homologene）。使用Mutation Taster、PolyPhen-2、PROVEAN、FATHMM、VEST3、MetaLR等在线生物信息学软件对基因突变进行致病性预测。通过蛋白质数据库（https://www.uniprot.org/）中提供的PROC基因（AF-P04070-F1.pdb）、SERPINC1基因（AF-P01008-F1.pdb）三维结构蛋白模型，应用PyMol软件分析突变型蛋白模型局部空间构型及分子间作用力的变化。

## 结果

一、凝血结果

家系1先证者的PC:A和PC∶Ag分别为57％和59.2％，其父亲PC∶A和PC∶Ag分别为54％和65.8％，家系成员其他相关结果均无明显异常；家系2先证者的PC∶A和其母亲的AT∶A均略偏低，分别为68％和78％，家系成员其余结果均在正常参考区间内（表1）。

**表1:** 2个抗凝血酶和蛋白C联合缺陷家系的凝血试验结果

家系成员	年龄（岁）	AT∶A（％）	PC∶A（％）	PS∶A（％）	AT∶Ag（mg/L）	PC∶Ag（％）	临床症状
家系1							
先证者（Ⅱ1）	17	109	57	106	294.4	59.2	DVT
父亲（Ⅰ1）	49	112	54	79	267.0	65.8	无症状
母亲（Ⅰ2）	46	89	126	79	320.9	95.1	无症状
家系2							
先证者（Ⅱ1）	42	116	68	93	253.3	107.3	DVT、PE
父亲（Ⅰ1）	63	104	72	113	253.3	132.5	无症状
母亲（Ⅰ2）	60	78	129	105	276.0	129.0	无症状

参考值		84～120	70～140	60～130	250.0～360.0	75.0～150.0	

**注** AT∶A：抗凝血酶活性；PC∶A：蛋白C活性；PS∶A：蛋白S活性；AT∶Ag：抗凝血酶抗原；PC∶Ag：蛋白C抗原；DVT：下肢静脉血栓形成；PE：肺栓塞

二、基因突变分析结果

基因检测结果发现，家系1先证者存在PROC基因第5外显子c.400+5G>A剪切位点突变和SERPINC1基因第5外显子c.883G>A杂合错义突变，分别导致PROC第5号内含子中插入13个碱基和SERPINC1的单个氨基酸的改变（p.Val295Met）；家系调查显示，2个突变位点分别来自其父亲和母亲。家系2先证者存在PROC基因第9外显子c.811C>T和SERPINC1基因第5外显子c.880C>T杂合错义突变，同样导致相应基因的单个氨基酸改变（p.Arg271Trp和p.Arg294Cys），突变位点同样分别来源于患者的父亲和母亲（图1）。

**图1:**
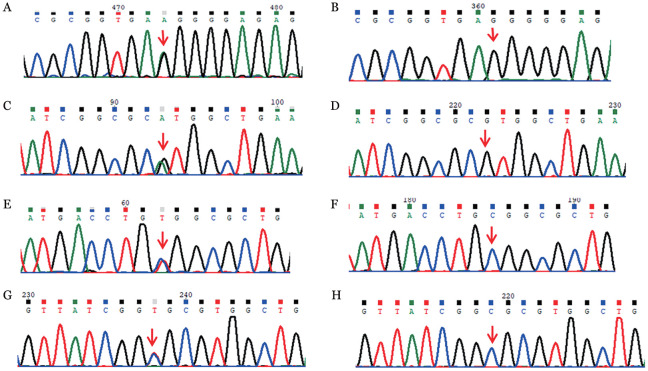
两个抗凝血酶和蛋白C联合缺陷家系基因测序结果（箭头所示为突变位点） **A、B** 分别为先证者1携带的PROC基因的c.400+5G>A剪切位点突变和其野生型；**C、D** 分别为先证者1携带的SERPINC1基因的c.883G>A杂合突变和其野生型；**E、F** 分别为先证者2携带的PROC基因的c.811C>T杂合突变和其野生型；**G、H** 分别为先证者2携带的SERPINC1基因的c.880C>T杂合突变和其野生型

三、同源性物种保守性分析结果

通过ClustalX-2.1-win软件对同源物种相应氨基酸保守性进行分析比对，结果表明PC的p.Arg271在7个同源物种间高度保守（图2A）；AT的p.Arg294和p.Val295在8个同源物种间亦高度保守（图2B）；表明以上3个位点氨基酸在其蛋白的结构形成和功能运用中起到重要作用。

**图2:**
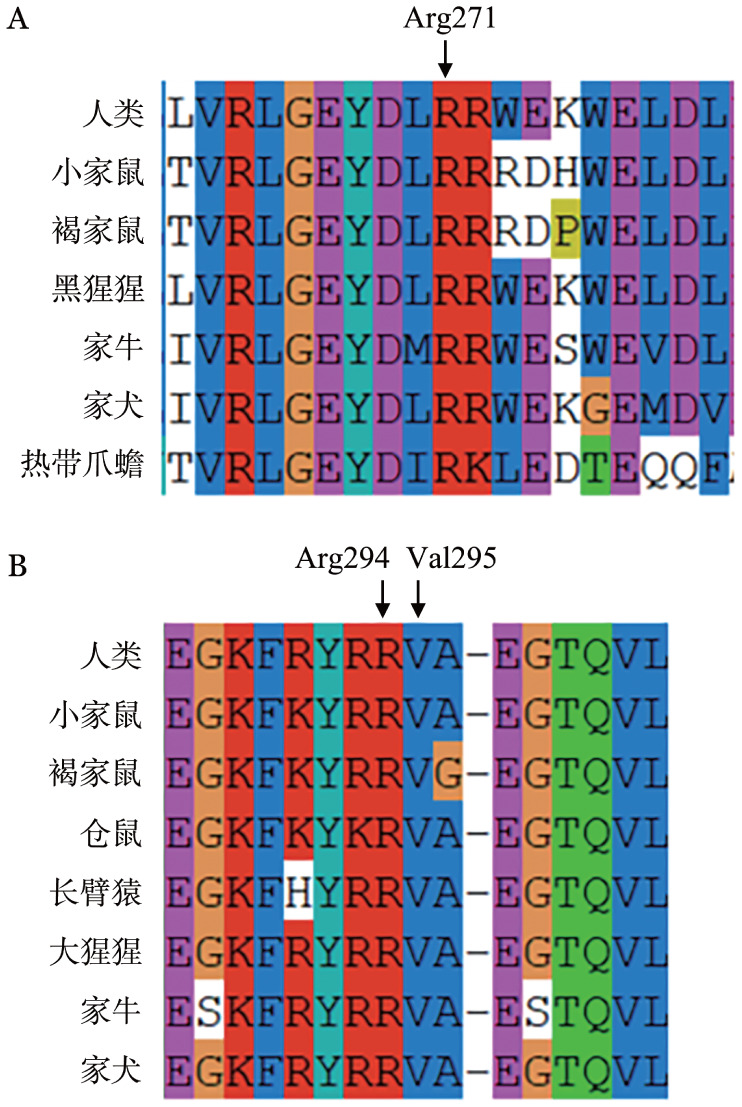
3个突变位点的保守性分析结果 **A** 蛋白C的氨基酸Arg271位点及其附近残基在同源性物种中的保守性；**B** 抗凝血酶的氨基酸Arg294和Val295位点及其附近残基在同源性物种中的保守性

四、在线生物信息学软件和蛋白结构模型分析结果

对于AT的c.883G>A突变，SIFT、CADD、FATHMM和PolyPhen-2的预测结果一致，将其分类为“有害的”和“可能有害的”；AT的c.880C>T突变也被VEST3、CADD、FATHMM和PolyPhen-2预测为“有害的”和“可能有害的”；MutationTaster、PolyPhen-2、PROVEAN等7个在线软件一致地将PROC c.811C>T突变分类为“致病性”，可影响相关蛋白质功能。运用PyMol蛋白质空间结构模型分析显示（图3），当Arg294突变为Cys294后，其侧链明显缩短，且与相邻Gly298氨基酸残基原有的氢键消失；而Val295突变为Met295时，后者延长的侧链可能改变分子之间的空间作用力；此外，当Arg271突变为Trp271后，与相邻Asp269氨基酸残基的3个氢键消失，且后者的2个苯环增加了分子之间的空间位阻。以上3个突变后的蛋白质结构模型分析结果均提示，可能导致蛋白质稳定性下降，影响蛋白质的功能。

**图3:**
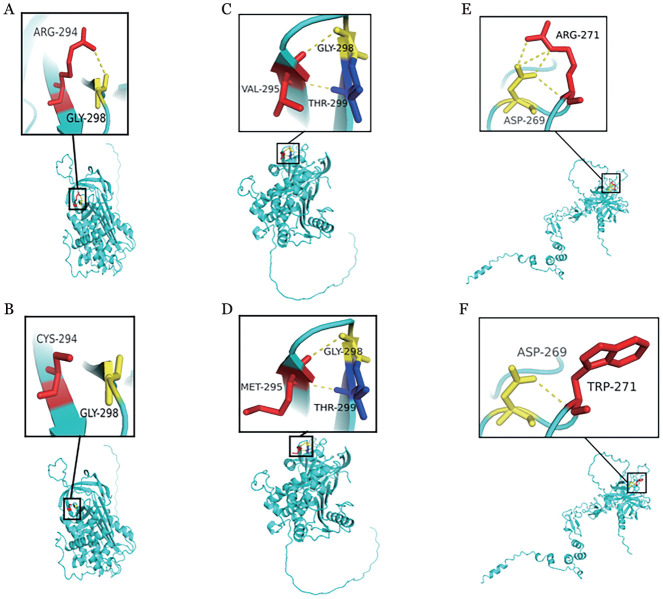
抗凝血酶和蛋白C突变蛋白空间结构模型变化（黄色虚线表示氢键） **A** SERPINC1p.Arg294野生型；**B** SERPINC1 p.Arg294Cys突变型；**C** SERPINC1 p.Val295野生型；**D** SERPINC1 p.Val295Met突变型；**E** PROC p.Arg271野生型；**F** PROC p.Arg271Trp突变型

五、凝血酶生成试验

与健康对照相比，4例突变携带者的TGT相关指标ETP和Peak较健康对照组均呈不同程度的增加，提示2个PC突变和2个AT突变均会导致血液高凝状态（图4）。当用5 nmol/L浓度的sTM处理后，PC活化并发挥抗凝功能，使得健康对照血浆凝血酶生成呈明显下降的趋势，ETP和Peak分别降至基线23.8％和37.6％，ETP抑制率为76.2％；而分别携带PC的c.400+5G>A和p.Arg271Trp突变携带者，凝血酶生成的下降趋势并不明显，ETP抑制率仅为13.9％和40.0％，提示基因突变导致PC抗凝功能明显下降（表2）。

**图4:**
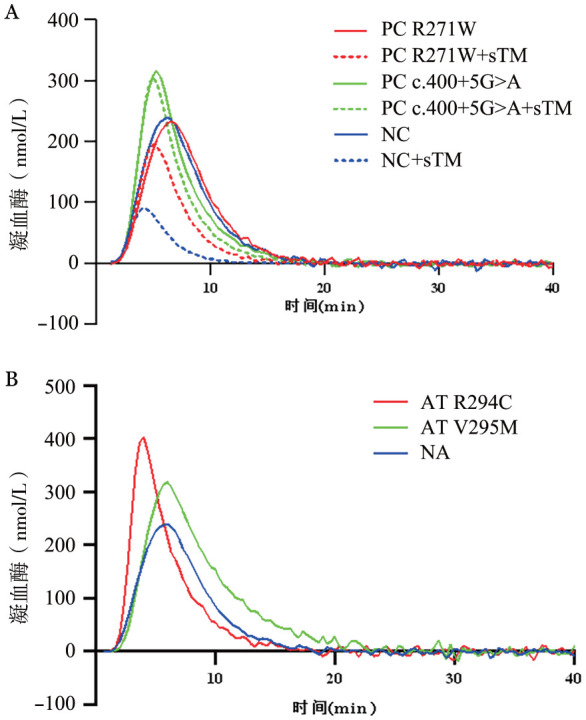
4个突变携带者的凝血酶生成试验曲线 **A** PC突变携带者的凝血酶生成及抑制试验曲线；**B** AT突变携带者的凝血酶生成试验曲线 **注** PC：蛋白C；AT：抗凝血酶；sTM：可溶性血栓调节蛋白；NC：正常健康对照

**表2:** 4例突变携带者的血浆凝血酶生成试验的结果

突变类型	ETP （nmol·L^−1^·min^−1^）	Peak （nmol/L）	STT （min）	ETP抑制率 （％）
PC 400+5G>A	1599.31	313.67	21.33	13.9
PC 400+5G>A+sTM	1376.1	303.81	19.67	
PC R271W	1512.21	233.19	23.33	40.0
PC R271W+sTM	898.75	196.8	18.67	
AT V295M	2272.69	316.01	27.5	–
AT R294C	1720.06	400.9	19.67	–
NC	1518.9	239.01	22.68	76.2
NC+sTM	360.8	89.79	17.16	

**注** ETP：内源性凝血酶生成能力；Peak：凝血酶生成峰值；STT：起始拖尾时间；ETP抑制率；PC：蛋白C；AT：抗凝血酶；NC：健康对照；sTM介导前后的凝血酶生成抑制比率

## 讨论

机体生理状态下，凝血与抗凝过程保持着动态平衡。当某些抗凝蛋白发生数量或质量缺陷时，易导致血栓形成。静脉血栓栓塞症（Venous thromboembolism,VTE）是由于遗传和环境因素等多种刺激因素之间的相互作用触发，遵从多重打击学说，机体主要的生理性抗凝物质（AT、PC和PS）缺乏是亚洲人的重要危险因素[6]。本研究中，先证者1为青少年，没有VTE的获得性危险因素，但出现下肢静脉、髂外静脉血栓形成致PE，通过易栓症基因Panel检测发现存在SERPINC1基因的c.883G>A（p.Val295Met）杂合错义突变和PROC基因的c.400+5G>A剪切位点突变，家系成员常规表型筛查仅发现先证者PC∶Ag略偏低（59％），其父亲的PC∶A和PC∶Ag轻度下降，分别为54％和65％；先证者2多年来数次出现VTE情况，未予重视，仅间断性口服抗凝药物治疗，最后一次出现PE后就诊，经易栓症基因Panel检测发现携带SERPINC1基因的c.880C>T（p.Arg294Cys）和PROC基因的c.811C>T（p.Arg271Trp）两个杂合错义突变。经过溶栓和抗凝治疗后2例患者的病情都明显好转，但考虑两例先证者均多次发生血栓，建议其进行个性化抗凝方案治疗。在随后的家系调查中，证实了2例先证者的基因突变均由父母遗传所致。

1965年，Egeberg等[7]首次报道了遗传性AT缺陷症作为血栓栓塞的危险因素，由于AT独特的抗凝机制和广泛的抗凝活性，轻度缺乏会增加血栓形成的风险，而严重缺乏会使静脉血栓形成的风险增加5～50倍[8]。值得注意的是，日本的一项深静脉血栓患者抗凝蛋白基因突变队列研究发现c.880C>T突变携带者的AT∶A在正常范围内[9]。Zeng等[10]在研究中也发现携带c.883G>A和c.880C>T突变的患者表现出AT∶A的正常和内源性凝血酶生成量的显著增高，且重组蛋白模型支持这些突变对AT表达和AT∶A的影响较小。我们的研究也发现类似的AT表型正常但内源性凝血酶生成增多的现象。由于Arg294和Val295位于β-折叠A的中间区域，靠近AT的反应中心环（Reactive center loop，RCL），虽然不直接参与RCL的功能，但其所在区域的结构变化可间接影响RCL的柔韧性和与靶标蛋白酶（如凝血酶、因子Ⅹa）的结合能力[11]。由此我们推断，突变导致的s1B末端残基的修饰对于AT蛋白的生成影响可能有限，但会损害AT与靶蛋白酶（凝血酶）的相互作用，导致抗凝血能力的降低。由于体外测定AT∶A时，检测试剂中存在过量肝素，肝素会大幅提升AT对底物的灭活作用，相当于弥补了突变导致的AT与底物作用的缺陷，使得测定结果在正常范围内。而生理条件下，体内并不会有大量肝素的存在，此时AT对底物的灭活作用仍处于减弱的状态，具有易栓倾向，这也就解释了AT∶A测定正常而两例先证者发生了VTE的原因。此外，本研究也给到另一个重要提示，对于表型正常但存在明显易栓倾向的无明显诱因性血栓栓塞症患者，易栓症基因筛查显得尤为重要。

PC的c.400+5剪切位点位于活化肽区域，靠近丝氨酸蛋白酶结构域的起始部分，这一区域在PC被凝血酶-TM复合物激活时被切割，释放活化肽，生成活化PC，通过灭活凝血因子Ⅴa和Ⅷa抑制凝血酶的生成，发挥抗凝作用[12]。先证者1的PC c.400+5G>A剪切位点突变可能会破坏原有的剪接方式，导致外显子跳跃[13]。多个报道[14]–[15]显示，该剪切位点突变为致病突变，可引起PC水平和功能异常。先证者2的p.Arg271Trp突变位于PC的丝氨酸蛋白酶结构域[16]，靠近253His-299Asp-402Ser催化三联体，该区域主要包括Ca^2+^结合环和自溶环，可通过激活钙依赖性相关肽来增加凝血酶-TM复合物的激活。Alsultan等[17]报道了2例p.Arg271Cys纯合突变新生儿患者，其突变重组蛋白在凝血酶-TM的激活率上存在显著缺陷，激活的效率降低了99％以上。我们的TGT抑制试验亦发现p.Arg271Trp突变携带者，其ETP抑制率仅为40.0％，提示基因突变可能影响了凝血酶-sTM的结合能力。蛋白模型分析显示，突变导致Arg271与相邻氨基酸Asp269之间3个氢键消失且新生成的2个苯环增加了分子之间的空间位阻，影响了PC蛋白催化结构域的完整性和稳定性，导致PC催化活性和血浆中PC水平的降低，可引起血栓风险增高。

AT和PC作为阻遏凝血酶转化纤维蛋白原的重要蛋白，因基因突变导致抗凝异常为血栓形成提供了条件。研究表明，在小鼠模型中，肝脏AT和PC生成的减少可能导致急性和严重静脉血栓形成[18]。在本研究中，携带AT或PC其中单个突变的患者父母未发现血栓史，而同时携带两个突变的患者在早期就患有反复的严重VTE，这表明父母患血栓事件的风险可能相对较低，而不同遗传风险因素的叠加进一步增加了VTE风险。由于PROC或SERPINC1基因突变个体形成血栓的风险差异很大，取决于多种因素相互作用，如长期制动、妊娠、外伤、手术、恶性肿瘤、吸烟、高龄、高血压、糖尿病和高血脂等[19]，因此，对于本研究2个家系成员的长期健康管理显得尤为重要。

综上所述，本研究通过对2个罕见AT和PC联合缺陷家系的临床特征及基因分析发现，PROC基因的c.400+5G>A、p.Arg271Cys和SERPINC1基因的p.Val295Met、p.Arg294Cys突变可能与2例先证者出现严重的VTE有关；采用在线生物信息学软件、Pymol蛋白分析软件、凝血酶生成试验，初步探讨了其致病机制，但具体致病机制有待进一步体外实验开展研究。

## References

[1] Zhang D, Sun B, Zhang X (2020). A novel SERPINC1 frameshift mutation in two antithrombin deficiency families[J]. Int J Lab Hematol.

[2] Lu Y, Giri H, Villoutreix BO (2020). Gly197Arg mutation in protein C causes recurrent thrombosis in a heterozygous carrier[J]. J Thromb Haemost.

[3] Bereczky Z, Gindele R, Fiatal S (2020). Age and origin of the founder antithrombin budapest 3(p.Leu131Phe) mutation; its high prevalence in the Roma population and its association with cardiovascular diseases[J]. Front Cardiovasc Med.

[4] Li L, Li J, Wu X (2023). Evaluation of prothrombotic risk of two PROC hotspot mutations (Arg189Trp and Lys193del) in Chinese population: a retrospective study[J]. Thromb J.

[5] Hemker HC, Giesen P, Al Dieri R (2003). Calibrated automated thrombin generation measurement in clotting plasma[J]. Pathophysiol Haemost Thromb.

[6] Tsuda H, Noguchi K, Oh D (2020). Racial differences in protein S Tokushima and two protein C variants as genetic risk factors for venous thromboembolism[J]. Res Pract Thromb Haemost.

[7] Egeberg O (1965). Inheried antithrombin deficiency causing thrombophilia[J]. Thromb Diath Haemorrh.

[8] Reda S, Müller J, Pavlova A (2021). Functional characterization of antithrombin mutations by monitoring of thrombin inhibition Kinetics[J]. Int J Mol Sci.

[9] Miyata T, Sato Y, Ishikawa J (2009). Prevalence of genetic mutations in protein S, protein C and antithrombin genes in Japanese patients with deep vein thrombosis[J]. Thromb Res.

[10] Zeng W, Hu B, Tang L (2017). Recurrent mutations in a SERPINC1 hotspot associate with venous thrombosis without apparent antithrombin deficiency[J]. Oncotarget.

[11] Rezaie AR, Giri H (2020). Anticoagulant and signaling functions of antithrombin[J]. J Thromb Haemost.

[12] Dahlbäck B, Villoutreix BO (2005). Regulation of blood coagulation by the protein C anticoagulant pathway: novel insights into structure-function relationships and molecular recognition[J]. Arterioscler Thromb Vasc Biol.

[13] Li X, Li X, Li X (2019). Genotypic and phenotypic character of Chinese neonates with congenital protein C deficiency: a case report and literature review[J]. Thromb J.

[14] Hoshi S, Hijikata M, Togashi Y (2007). Protein C deficiency in a family with thromboembolism and identified gene mutations[J]. Intern Med.

[15] Tang X, Zhang Z, Yang H (2022). Clinical and genetic features of Chinese pediatric patients with severe congenital protein C deficiency who first presented with purpura fulminans: A case series study and literature review[J]. Thromb Res.

[16] 曾 蔓霖, 杨 丽红, 邹 安庆 (2023). 两个遗传性蛋白C缺陷症家系的临床特征与基因突变分析[J]. 中华血液学杂志.

[17] Alsultan A, Gale AJ, Kurban K (2016). Activation-resistant homozygous protein C R229W mutation causing familial perinatal intracranial hemorrhage and delayed onset of thrombosis[J]. Thromb Res.

[18] Ünlü B, Heestermans M, Laghmani EH (2024). The effects of an aggressive breast tumor on thrombosis after antithrombin downregulation in a hypercoagulable mouse model[J]. Thromb Res.

[19] 徐 琦煜, 杨 丽红, 谢 海啸 (2022). 不同家系遗传性蛋白C缺陷症12例临床表型和基因突变分析[J]. 中华血液学杂志.

